# Stimulation of TLR4 by LMW-HA Induces Metastasis in Human Papillary Thyroid Carcinoma through CXCR7

**DOI:** 10.1155/2013/712561

**Published:** 2013-12-02

**Authors:** Shipeng Dang, Yongde Peng, Lei Ye, Yanan Wang, Zhongqing Qian, Yuqing Chen, Xiaojing Wang, Yunzhi Lin, Xiaomei Zhang, Xiyan Sun, Qiong Wu, Yiji Cheng, Hong Nie, Min Jin, Huanbai Xu

**Affiliations:** ^1^Institute of Health Sciences, Shanghai Institutes for Biological Sciences, Chinese Academy of Sciences & Shanghai Jiao Tong University School of Medicine (SJTUSM), 225 South Chongqing Road, Shanghai 200025, China; ^2^Department of Endocrinology and Metabolism, Shanghai Jiaotong University Affiliated First People's Hospital, 100 Haining Road, Shanghai 200080, China; ^3^Department of Endocrinology and Metabolism, Ruijin Hospital, SJTUSM, 197 Ruijin 2nd Road, Shanghai 200025, China; ^4^Shanghai Institute of Immunology, Institutes of Medical Sciences, SJTUSM, 280 South Chongqing Road, Shanghai 200025, China; ^5^Department of Endocrinology and Metabolism, The First Affiliated Hospital of Bengbu Medical College and Anhui Clinical and Preclinical Key Laboratory of Respiratory Disease, The First Affiliated Hospital of Bengbu Medical College, 287 Changhuai Road, Bengbu 233004, China; ^6^Cancer Hospital, HeFei Institutes of Physical Science, Chinese Academy of Science, 350 Shushan Lake Road, HeFei 230031, China

## Abstract

In inflammatory sites, high molecular weight hyaluronan fragments are degraded into lower molecular weight hyaluronan fragments (LMW-HA) to regulate immune responses. However, the function of LMW-HA in PTC progression remains to be elucidated. In this study, we found that receptor of LMW-HA, TLR4, was aberrantly overexpressed in PTC tissues and cell line W3. Exposure of W3 cells to LMW-HA promoted cell proliferation and migration via TLR4. Knockdown of TLR4 has provided evidence that TLR4 is essential for LMW-HA-induced CXCR7 expression, which is responsible for LMW-HA-induced proliferation and migration of W3 cells. In tumor-bearing adult nude mice, stimulation of LMW-HA on W3 cells promotes CXCR7 expression in tumor masses (*P* = 0.002) and tumor growth (*P* < 0.001). To further confirm our findings, we investigated the clinicopathologic significance of TLR4 and CXCR7 expression using immumohistochemistry in 135 human PTC tissues and 56 normal thyroid tissue samples. Higher rates of TLR4 (53%) and CXCR7 (24%) expression were found in PTC tissues than in normal tissues. Expression of TLR4 or CXCR7 is associated with tumor size and lymph node metastasis. Therefore, LMW-HA may contribute to the development of PTC via TLR4/CXCR7 pathway, which may be a novel target for PTC immunomodulatory therapy.

## 1. Background

Papillary thyroid cancer (PTC) is the most prevalent thyroid cancer and represents 70 to 80% of all thyroid cancers [1]. Incidence of thyroid cancer has increased rapidly in the past 15 years the increase in incidence is almost exclusively attributable to papillary thyroid cancer [2]. Metastasis is the most important biological characteristic of PTC. That is, PTC has a tendency to spread into lymphatic channels and metastasize to regional lymph nodes at a high frequency.

It is known that inflammation plays critical roles in the development of cancers including PTC. It has been reported that T cells, B cells, and NK cells are frequently found within and surrounding primary thyroid tumor [3]. French et al. revealed that PTC patients with tumor-associated lymphocytic infiltration presented more aggressive disease when compared with patients with concurrent thyroiditis or without lymphocytic infiltration [4], suggesting the presence of a local inflammatory response in PTC.

The extracellular matrix is important for tumor cell behavior. Hyaluronan (HA) is a polysaccharide normally expressed in the extracellular matrix of connective, neural, and epithelial tissues [5]. Under physiological conditions, HA is primarily distributed in connective tissue with many other proteins to form a large and complicated network that maintains the space between cells [5, 6]. The native, high molecular weight form (HMW-HA) is composed of repeating disaccharide units of N-acetylglucosamine and glucuronic acid (4 × 10^2^ to 2 × 10^4^ kDa) and is synthesized on the surface of a variety of cells. HMW-HAs are space-filling molecules that hydrate tissues they not only play a role in cell adhesion but also are antiangiogenic, anti-inflammatory, and immunosuppressive [7]. HMW-HA can be degraded into lower molecular weight fragments (LMW-HA) by hyaluronidases, whose expression is elevated during the process of inflammatory responses, tumor development, and tissue injury [3, 8]. Recent study shows that LMW-HA, not the native HMW-HA, can initiate inflammatory responses in dendritic cells in skin transplant rejection [9]. It has been reported that there are three receptors of LMW-HA, TLR2, TLR4 and CD44. Binding of LMW-HA to these receptors could activate immune cells and promote the production of different cytokines by macrophages, activated dendritic cells, and T cells [10–13].

Tumor cells have also been shown to produce hyaluronidases, which lead to HA degradation in the tumor surrounding environment [14]. Voelcker et al. suggested that LMW-HA in melanoma might promote tumor invasiveness by inducing MMP and cytokine expression, partly in a TLR4-dependent manner [15], providing new insights into the relationship between cancer and innate immunity. Moreover, Bourguignon et al. suggested that LMW-HA played an important role in CD44-TLR-associated AFAP-110-actin interaction and MyD88-NF-*κ*B signaling, which was required for tumor cell behaviors [16]. Besides, LMW-HA inhibits colorectal carcinoma growth by decreasing tumor cell proliferation and stimulating immune response [17]. Bohm et al. suggested that strong stromal HA staining intensity was related to the progression and unfavorable outcome in differentiated thyroid carcinoma (DTC) patients including PTC [18]. However, little else is known about the function of LMW-HA in PTC development.

Key molecules such as chemokines/chemokine receptors not only attract leukocytes to local inflammatory sites but also directly enhance the survival, proliferation, and migration of tumor cells. The chemokine CXCL12 (also called stromal-derived factor-1) is an important chemokine that binds CXCR4/CXCR7, playing important roles in promoting tumor cell proliferation and migration [19].

In this study, we investigated the roles of LMW-HA in the progression of PTC. We found that TLR4 was aberrantly overexpressed in PTC. Moreover, stimulation of LMW-HA induced CXCR7 expression in PTC cells via TLR4 signaling to promote the proliferation and migration of PTC cells. Furthermore, tumor-bearing mice and clinicopathology were used to verify that the LMW-HA/TLR4/CXCR7 pathway may be critical during the development of PTC, indicating LMW-HA as a possible novel immunomodulatory therapy target for PTC treatment.

## 2. Material and Methods

### 2.1. Cell Lines

Human PTC cell line K1 was purchased from the American Type Culture Collection. Cell lines, W3, and TPC1 were kind gifts from Dr. Robert Gagel (MD Anderson Cancer Center, University of Texas, USA). All cells were cultured at 37°C and 5% CO_2_. K1 cells were maintained in DMEM : Ham's F12 : MCDB 105 (2 : 1 : 1) (Invitrogen) and supplemented with 10% fetal bovine serum (FBS) (Invitrogen) and 100 *μ*g/mL streptomycin (Invitrogen) and 100 U/mL penicillin (Invitrogen). W3 cells were maintained in RPMI 1640 (Invitrogen) with 10% FBS (Invitrogen) and 100 *μ*g/mL streptomycin (Invitrogen) and 100 U/mL penicillin (Invitrogen). TPC1 cells were maintained in DMEM (Invitrogen) with 10% FBS (Invitrogen) and 100 *μ*g/mL streptomycin (Invitrogen) and 100 U/mL penicillin (Invitrogen).

### 2.2. Animals and Tumor Model

Female nude mice (6–8 week) were purchased from the Shanghai Laboratory Animal Center at the Chinese Academy of Sciences and housed in a specific pathogen-free facility at the Shanghai Jiao Tong University School of Medicine. All animal procedures were approved by the Animal Welfare & Ethics Committee of Shanghai Jiao Tong University School of Medicine. Tumors were xenografted onto the left flank of mice through a subcutaneous injection of 6 × 10^6^ W3 cells in 50 *μ*L of phosphate buffered saline (PBS). Mice were intratumorally injected with LMW-HA (4,900 Da, JIANGSU HAIHUA BIOTECH CO, China, 400 *μ*g/kg) or the same volume of DMSO every other day. Tumors were measured with a caliper every fourth day and tumor volumes were calculated using the formula (length × width^2^)/2. When maximum diameters of tumors reached about 1.0 cm, mice were euthanized. Tumors were removed and weighed. CXCR7 expression in tumor tissues was analyzed by immunohistochemistry.

### 2.3. Patients and Specimens

This study was approved by the Medical Ethics Committee of The First Affiliated Hospital of Bengbu Medical College and all works were conducted in accordance with the Declaration of Helsinki. All participants gave informed written consent before participating in this study. PTC samples were collected from 135 patients undergoing curative-intent surgery at the Department of Surgery, The First Affiliated Hospital of Bengbu Medical College between 2001 and 2011. There were also 56 normal thyroid tissue samples adjacent to papillary thyroid carcinoma (used as controls). The histologic sections were reviewed by two expert pathologists to verify the histologic diagnosis. None of the patients had received any preoperative treatment. Tumors were staged according to the American Joint Committee on Cancer (AJCC) pathologic tumor-node-metastasis (TNM) classification.

### 2.4. Western Blot Analysis

PTC cell lines were lysed with RIPA Lysis Buffer (Beyotime, China) supplemented with protease inhibitor Cocktail (AppliChem, Germany). Protein concentration in the postnuclear lysates was measured by BCA Protein Assay Kit (Beyotime, China) and equal amounts of protein lysates (60 *μ*g) were loaded on 10% SDS-PAGE. Gels were transferred to nitrocellulose using iBlot Dry Blotting System (Invitrogen, USA). Filters were blocked with 5% dry skimmed milk and blotted with the specific primary antibodies: mouse monoclonal antibody to TLR4 (Abcam, England), TLR2 (eBioscience, USA), or CD44 (eBioscience, USA). Blots were then incubated with the appropriate HRP-conjugated secondary antibody (Beyotime, China), and signals were detected by the WestPico chemiluminescence system (Pierce). Filters were stripped for 10 min with ReBlot Plus Strong Antibody Stripping Solution (Millipore).

### 2.5. Flow Cytometric Assay

For *in vitro* studies of CXCR7 and CXCR4 expression, all cell lines were cultured in medium containing 2% FBS. After 12 h, W3 cells were treated with LMW-HA for 24 h. Then cells were trypsinized and incubated for 1 h with a monoclonal anti-human CXCR7 antibody (R&D systems, USA) or CXCR4 antibody (BD Biosciences, USA) and analyzed using a flow cytometry (BD Aria).

### 2.6. Cell Transfection

siRNA sequences (TLR4: 5′-GAGCCGCUGGUGUAUCUUU-3′, TLR4-KD; CXCR7: 5′-CCGUUCCCUUCUCCAUUAU-3′, CXCR7-KD; Scrambled siRNA: 5′-AGGACTGAGTGTACCGTCT-3′, Scram) were designed into shRNA and inserted into pGPU6/GFP/Neo vector (GenePharma, Shanghai, China) under U6 promoter. Cells resistant to G418 (800 *μ*g/mL) were selected and expanded for further study. The depletion of endogenous TLR4 or CXCR7 by the shRNA was confirmed by immunoblot.

### 2.7. Cell Proliferation Assay

W3 cells were cultured in 96-well plates at an initial density of 2,000 cells per well, in 100 *μ*L of 1% FBS-medium with or without addition of LMW-HA (100 ng/mL) for indicated times. Cell proliferation was determined using a WST-1 Kit (Beyotime, China). Each experimental condition was sampled in triplicate and the experiments were repeated three times.

### 2.8. Apoptosis Assay

W3 cells were incubated in 1% FBS-medium with or without addition of LMW-HA for 24 hours and incubated for 30 min at room temperature with 0.5 mg/mL propidium iodide (PI, eBioscience, USA) and annexin V-FITC (eBioscience, USA). Then cells were analyzed with flow cytometry. Each experiment was repeated three times.

### 2.9. Migration Experiments

W3 cells were resuspended in 1% FBS-medium at 5 × 10^5^ cells/mL and seeded into the upper chambers of Transwell inserts (Millipore). 1% FBS-medium was added to the lower chambers, with or without addition of CXCL12 (100 ng/mL). After incubation with LMW-HA, the nonmigrated cells were removed from the upper surface of the filters, and the migrated cells, adherent to the lower surface, were counted (Ten high-power fields/well). Each experiment was repeated three times.

### 2.10. Immunohistochemistry Assay

Sections were subjected to routine deparaffination and rehydration. Antigen retrieval was achieved by microwaving in 0.01 mol/L citrate buffer for 10 min and then cooled for 30 min. The endogenous peroxidase activity was inhibited by incubation with 3% hydrogen peroxide in methanol for 20 min and nonspecific binding was blocked with 5% bovine serum albumin in PBS at room temperature. After three PBS washes, the specimens were incubated overnight at 4°C with murine anti-human TLR4 and CXCR7 monoclonal antibodies. After incubation with rat anti-mouse-IgG2b-horseradish peroxidase, signal was developed with 3, 30-diaminobenzidine tetrahydrochloride in Tris-HCl buffer (pH 7.6) containing 0.02% hydrogen peroxide. The sections were then counterstained with hematoxylin and mounted. Negative controls were performed by replacing the primary antibody with nonspecific IgG at the same concentration.

### 2.11. Interpretation and Evaluation of Immunohistochemical Results

Immunostaining was independently examined by two clinical pathologists who were unaware of the patient outcome. For each sample, five high-power fields (100×) were randomly selected. Staining intensity and percentage of positive tumor cells were assessed. The extent of the staining was categorized into five semiquantitative classes based on the percentages of positive tumor cells: 0 (<5% positive cells), 1 (6–25% positive cells), 2 (26–50% positive cells), 3 (51–75% positive cells), and 4 (>75% positive cells). The intensity of cytoplasmic and membrane staining was also determined semiquantitatively on a scale of 0–3 as follows: 0 (negative), 1 (weakly positive), 2 (moderately positive), and 3 (strongly positive). A consensus score was assigned for each section after discussion and careful review of all slides by the two pathologists. Multiplication of the intensity and the percentage scores gave rise to the final staining score: negative (0), + (1–4), ++ (5–8), and +++ (9–12). For statistical analysis, tumors having a final staining score of negative or +, which showed a weak or moderate/strong immunoreactivity, were grouped into a low expression group and were compared to tumors with scores of ++ or +++ as the high expression group.

### 2.12. Statistical Analysis

Differences were evaluated using the Statistical Package for Social Science software (version 16.0, SPSS Inc., Chicago, IL). The association of staining intensity with clinicopathologic patterns was assessed with the Chi square test and two-sided Fisher's exact test to determine the significance of the difference between the covariates. All measurement data are presented as mean ± SEM. Statistical significance was evaluated by one-way ANOVA, followed by the least significant difference (LSD) test. *P* values <0.05 were considered to be statistically significant.

## 3. Results

### 3.1. TLR4 Is Highly Expressed in PTC Tissues and Cell Lines

**Figure 1:**
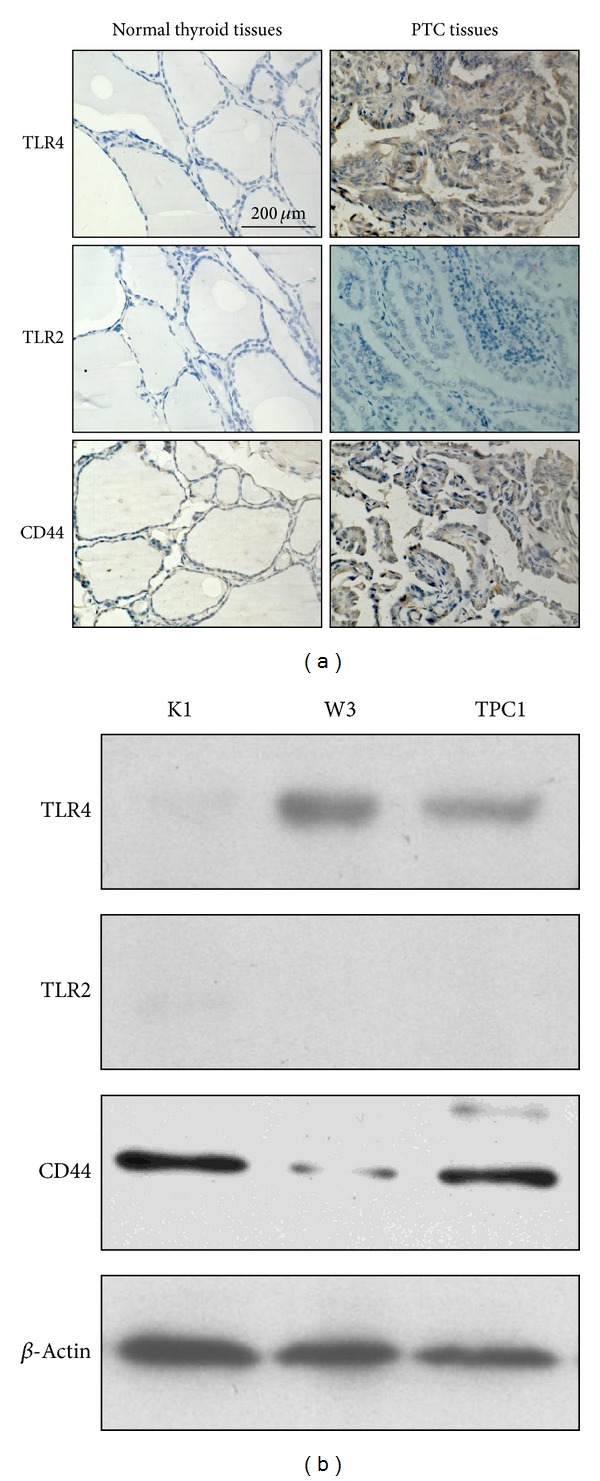
Increased TLR4 expression in PTC tissues and cell line W3. (a) Representative examples of IHC staining analyses of TLR4, TLR2, and CD44 in human normal thyroid tissues and PTC tissues (original magnification 400×). (b) Immunoblot analysis of the expression of TLR4, TLR2, and CD44 on 3 human PTC cell lines. Representative results are shown.

It has been shown that lymphocytic infiltration presented in or around PTC tissues, which mediated local inflammatory responses and affected the progression of PTC [4]. In sites of inflammation, HMW-HA may be degraded into LMW-HA, which in return activates immune responses [3, 11]. It has been reported that strong stromal HA staining intensity is related to progression and unfavourable outcome in thyroid carcinoma patients [18]. To investigate whether LMW-HA played a role in the development of PTC, we assessed the expression levels of three LMW-HA receptors, TLR2, TLR4, and CD44, in PTC tissues and three different human PTC cell lines (K1, W3, and TPC1). Immunohistochemistry analysis showed that TLR4 was highly expressed in PTC tissues compared to normal thyroid tissues (Figure 1(a), Table 1, *P* < 0.001); TLR2 was virtually undetectable (<10% of cells) in both normal thyroid tissues and PTC tissues; CD44 was expressed in most of normal thyroid tissues (51 of 56) and PTC tissues (128 of 135) (data not shown). These data demonstrate that TLR4 is aberrantly overexpressed in PTC, suggesting that LMW-HA/TLR4 may participate in the development of PTC. At the same time, western blot analysis indicated that TLR4 was highly expressed on W3 cells, moderately expressed on TPC1 cells, and low or negatively expressed on K1 cells; CD44 was weakly expressed on W3 cells and strongly expressed on K1 and TPC1 cells; while TLR2 expression could not be detected on three of these cell lines (Figure 1(b)). As CD44 expression did not show difference between normal thyroid tissues and PTC tissues, TLR4^high^CD44^low^ PTC cell line, W3 cells were chosen for this study thereafter to exclude the possible effects of LMW-HA/CD44 signal on tumor progression.

### 3.2. LMW-HA Promotes Proliferation and Migration of W3 Cells via TLR4

**Figure 2:**
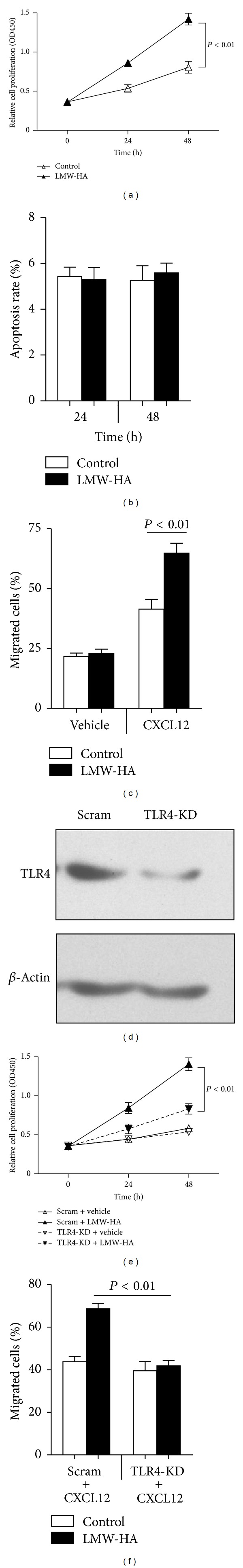
LMW-HA promotes W3 cell proliferation and migration via TLR4. (a) W3 cells were seeded into 96-well plates (2,000 cells/well) and treated with or without LMW-HA. Cell proliferation was analyzed with WST-1 Kit. Data are mean ± SEM for three independent experiments. (b) W3 cells were treated with or without LMW-HA for 24 hours and stained with annexin V and PI. Data are mean ± SEM for three independent experiments. (c) W3 cells were seeded into the upper chambers of transwell inserts treated with or without LMW-HA and in the presence or absence of CXCL12 in the lower chambers. Migrated cells were determined. Data are mean ± SEM for three independent experiments. (d) W3 cells were transfected with scrambled shRNA (Scram) or TLR4 shRNA-expressing constructs (TLR4-KD) and subjected to immunoblot analysis. (e) Scram-W3 cells and TLR4-KD W3 cells were seeded into 96 well plates and treated with or without LMW-HA (100 *μ*g/mL) for 24 h; cell proliferation was analyzed. Data are mean ± SEM for three independent experiments. (f) Scram-W3 cell and TLR4-KD W3 cell migration to CXCL12 treated with or without LMW-HA was determined. Data are mean ± SEM for three independent experiments.

Then we treated W3 cells with LMW-HA and determined the effects of LMW-HA on W3 cell apoptosis, proliferation, and migration. Stimulation of LMW-HA significantly enhanced the proliferation of W3 cells (Figure 2(a)) but did not induce apoptosis of W3 cells (Figure 2(b)). Moreover, LMW-HA significantly promoted the migration of W3 cells in the presence of CXCL12 (Figure 2(c)). Next we investigated if LMW-HA promotes proliferation and migration of W3 cells via the receptor, TLR4. When TLR4 expression was knocked down by shRNA (Figure 2(d)), LMW-HA induced proliferation and migration of W3 cells were mostly abolished (Figures 2(e) and 2(f)). In addition, we further stimulated TLR4 negative K1 cells with LMW-HA, and the data showed that LMW-HA did not promote the proliferation and migration of K1 cells (see Supplemental Figures (a) and (b) available online at http://dx.doi.org/10.1155/2013/712561). Collectively, these data suggest that LMW-HA promotes the proliferation and migration of W3 cells through activating TLR4 signal pathway.

### 3.3. LMW-HA Elevates CXCR7 Expression to Promote Proliferation and Migration of W3 Cells

**Figure 3:**
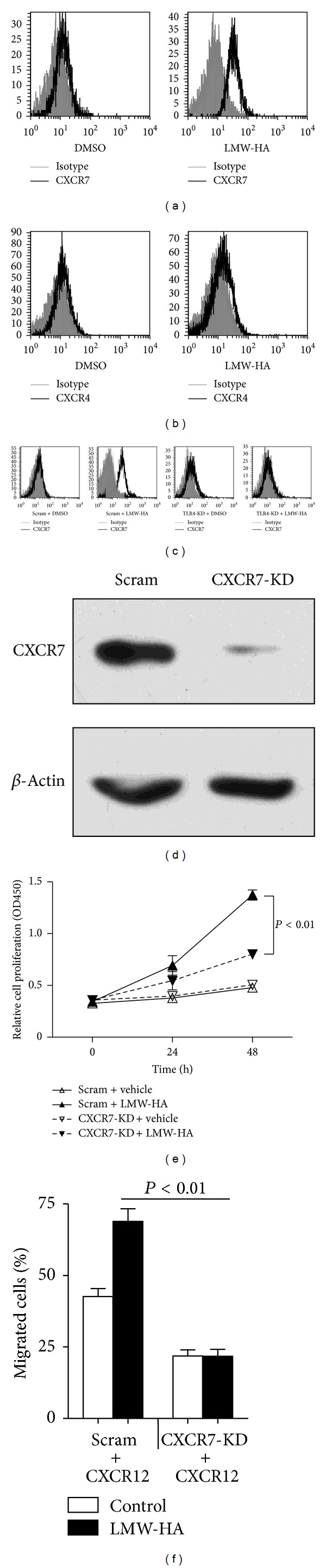
LMW-HA upregulates CXCR7 to promote W3 cell proliferation and migration. ((a)-(b)) W3 cells were incubated with LMW-HA (100 *μ*g/mL) for 24 h; representative flow cytometric analysis of CXCR7 (a) or CXCR4 (b) expression was shown. (c) Scram-W3 cells and TLR4-KD W3 cells were treated with or without LMW-HA (100 *μ*g/mL) for 24 h; representative flow cytometric analysis of CXCR7 expression was shown. (d) W3 cells were transfected with scrambled shRNA (Scram) or CXCR7 shRNA-expressing constructs (CXCR7-KD), and subjected to immunoblot analysis. (e) Scram-W3 cells and CXCR7-KD W3 cells were seeded into 96 well plates (2,000 cells/well) and treated with or without LMW-HA. Cell proliferation was analyzed. Data are mean ± SEM for three independent experiments. (f) Scram-W3 cell and CXCR7-KD W3 cell migration to CXCL12 treated with or without LMW-HA was determined. Data are mean ± SEM for three independent experiments.

To determine how LMW-HA promotes proliferation and migration of W3 cells, downstream molecules of TLR4 signal pathway, CXCR4 and CXCR7 expression were examined in W3 cells treated with or without LMW-HA. Neither CXCR4 nor CXCR7 was expressed in W3 cells, whereas exposing W3 cells to LMW-HA induced significant CXCR7 expression (Figure 3(a)). In contrast to CXCR7 expression alterations, LMW-HA had no effect on CXCR4 expression in W3 cells (Figure 3(b)). To investigate the role of TLR4 in LMW-HA-mediated CXCR7 expression, we knocked down TLR4 in W3 cells. Then LMW-HA-mediated CXCR7 expression was totally inhibited (Figure 3(c)). Next we assessed whether upregulation of CXCR7 expression was responsible for LMW-HA induced proliferation and migration of W3 cells. We found that knockdown of CXCR7 (Figure 3(d)) indeed blocked the proliferation and migration alterations in W3 cells induced by LMW-HA (Figures 3(e) and 3(f)). Moreover, LMW-HA also did not upregulate CXCR7 expression in TLR4 negative K1 cells. Taken together, these findings suggest that LMW-HA/TLR4-induced CXCR7 expression significantly promotes PTC cell proliferation and migration.

### 3.4. Stimulation of W3 Cells by LMW-HA Promotes Tumor Growth in Adult Nude Mice

**Figure 4:**
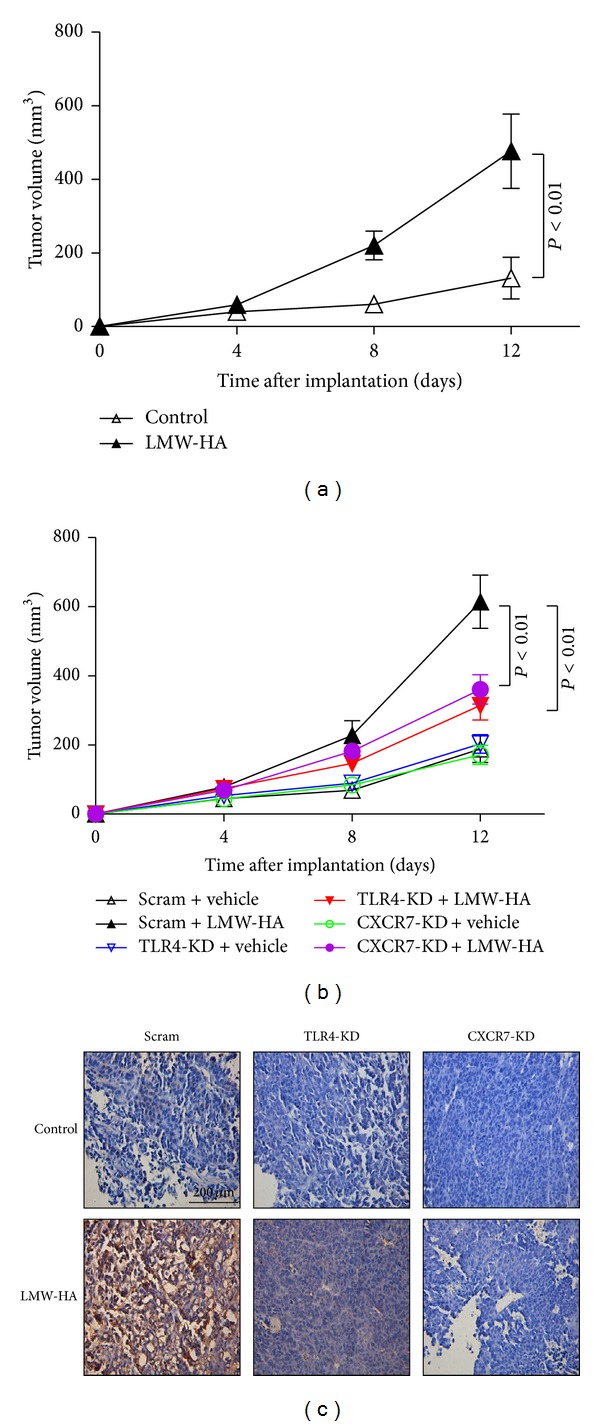
LMW-HA enhances the tumorigenicity of W3 cell line via TLR4/CXCR7 pathway. (a) W3 cells (6 × 10^6^ cell/mouse) were injected subcutaneously into the flanks of nude mice, and mice were intratumorally injected with LMW-HA (400 *μ*g/kg) or the same volume of DMSO every other day. Tumors were measured with a caliper every fourth day. When tumor maximum diameter reached about 1.0 cm, mice were euthanized and tumors were removed and weighed. The volumes of the tumor masses formed in LMW-HA treatment groups and control treatment groups were determined. (b) Scram-W3 cells, TLR4-KD W3 cells, and CXCR7-KD W3 cells were injected subcutaneously into the flanks of nude mice, and mice were intratumorally injected with LMW-HA (400 *μ*g/kg) or the same volume of DMSO every other day. Tumor volumes were determined and shown. (c) CXCR7 expression of tumors was analyzed by immunohistochemistry (original magnification 400×). These results were representative of three independent experiments.

To substantiate the effects of LMW-HA on tumor growth, female BALB/c nude mice were subcutaneously injected with W3 cells and treated with or without LMW-HA. The volumes of the tumor masses formed in LMW-HA treatment groups were larger than those of tumors from the control treatment groups (Figure 4(a)). To determine the effect of LMW-HA/TLR4/CXCR7 pathway on tumor growth *in vivo*, TLR4 or CXCR7 was inhibited with shRNA in W3 cells. Tumor growth was substantially inhibited in TLR4-KD and CXCR7-KD group in contrast to scrambled siRNA group in the presence of LMW-HA (Figure 4(b)), suggesting that LMW-HA may promote growth of W3-derived PTC model tumors *in vivo* through TLR4/CXCR7 pathway. Then immunohistochemistry analysis demonstrated that CXCR7 expression was significantly higher in tumor masses treated with LMW-HA in contrast to that of control treatment groups (Figure 4(c); Table 2, *P* = 0.002). Knockdown of TLR4 inhibited LMW-HA-induced expression of CXCR7 in tumor masses, indicating that CXCR7 was induced by LMW-HA in tumor tissue through TLR4 and might play important roles in tumorigenicity.

### 3.5. Expression of TLR4 or CXCR7 Is Associated with Tumor Size and Lymph Node Metastasis

**Figure 5:**
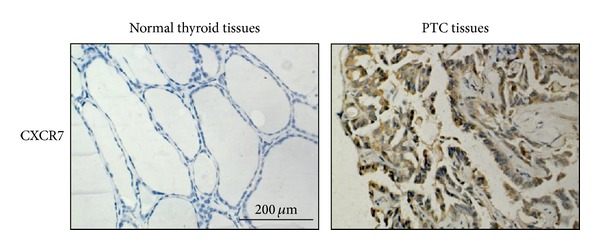
Increased expression of CXCR7 in PTC tissues. Representative examples of immunohistochemical staining of CXCR7 in normal thyroid tissues and PTC tissues (original magnification 400×). Representative results are shown.

To further determine whether LMW-HA/TLR4/CXCR7 pathway plays a role in PTC progression, we investigated the clinicopathologic significance of TLR4 and CXCR7 expression using immunohistochemistry in human PTC tissues. TLR4 and CXCR7 exhibited mostly cytoplasmic and plasmalemmal staining in carcinoma tissues (Figures 1(a) and 5). Normal tissue adjacent to tumor cells showed negative or occasionally weak staining that was mostly cytoplasmic (Figures 1(a) and 5). The differences in expression of the two molecules between carcinoma tissues and normal thyroid tissues were all found to be statistically significant (Table 1; TLR4, *P* < 0.001; CXCR7, *P* < 0.001). As shown in Table 1, tumor size tended to be larger in cases with high rather than low expression of TLR4 (*P* < 0.001) and CXCR7 (*P* < 0.001). There is a statistically significant correlation between TNM stage and TLR4 expression (*P* < 0.001) or CXCR7 expression (*P* = 0.005). The increased expression is significantly associated with advanced histological grade (TLR4, *P* = 0.001; CXCR7, *P* = 0.002). At the same time, the incidence of lymph node metastasis tended to be higher in patients with PTC with high rather than low expression of TLR4 (*P* < 0.001) or CXCR7 (*P* < 0.001). In addition, the incidence of distant metastasis tended to be higher in patients with PTC with high rather than low expression of TLR4 (*P* = 0.039) or CXCR7 (*P* < 0.001). There were no statistically significant differences in these molecules with regard to patient age and sex. All these data indicate that expression of TLR4 or CXCR7 is associated with PTC tumor size and lymph node metastasis.

## 4. Discussion 

Metastases, rather than primary tumors, are responsible for most cancer deaths. This process requires tumor cells to acquire the ability of proliferation, antiapoptosis, migration, and invasion. The presence of central neck lymph node metastases in PTC is known as an independent risk factor for recurrence. Our data showed that LMW-HA induced CXCR7 upregulation in PTC cells through TLR4 signaling, which promoted PTC cell line W3 proliferation and migration. Likewise, LMW-HA could also promote W3 cell proliferation in nude mice. Furthermore, higher rates of TLR4 and CXCR7 expression were found in human PTC tissues than in normal thyroid tissues, indicating that expression of these two molecules is associated with increased carcinoma growth and metastasis potential in human PTC.

In sites of inflammation or tissue injury, HA, ubiquitous in the extracellular matrix, is broken down into LMW-HA that has been reported to activate immunocompetent cells. For instance, it induces inflammatory chemokine and cytokine expression in macrophages [20]. Black et al. demonstrated a novel CD44 and MyD88 independent pathway for HA fragments to activate macrophage production of interferon-*β* via TLR4-TRIF-TBK1-IRF3 [21]. Stimulation of TLRs by LMW-HA induces self-defense mechanisms in vaginal epithelium [22]. LMW-HA increases the self-defense of skin epithelium by induction of *β*-defensin 2 via TLR2 and TLR4 [13]. LMW-HA and HMGB1 act as innate immune cytokine-like signals with the potential to modulate chondrocyte differentiation and function in OA progression via MyD88-dependent TLR2/TLR4 signaling [23]. Recently, LMW-HA has been shown to be associated with tumor invasiveness and metastasis [24]. Our data in this study have unraveled the crucial mechanisms underlying the promoting effect of inflammation-derived-LMW-HA signaling on the metastatic potential of PTC cells. A marked increase of CXCR7 expression was induced in a TLR4 positive PTC cell line W3, in response to LMW-HA. Knockdown of TLR4 in W3 cells has provided evidence that TLR4 is essential for LMW-HA-induced CXCR7 expression. Simultaneously, we established LMW-HA-W3 tumor-bearing mice model to further determine the function of the LMW-HA/TLR4/CXCR7 pathway in PTC.

CD44 is a primary cell-surface HA receptor. Binding of HA to CD44 plays roles in cell adhesion, immune responses, and tumor development. Although it has been reported that HA-CD44 signaling promotes the progression of several cancers, such as breast cancer [25], colorectal carcinoma [17], fibrosarcoma [26], and glioblastoma multiforme [27], in this study we found that CD44 was overexpressed in both normal tissue and PTC tissue and overexpression of CD44 was not relevant to progression of PTC. Further *in vitro* study may be needed to investigate the effect of CD44 on the proliferation and migration of PTC cell lines.

TLR4 expressed on tumor cells has been found to contribute to tumor progression by promoting tumor cell proliferation, apoptosis resistance, and tumor evasion from immune attack [28, 29]. LPS was released from the damaged cells or from bacteria in tumor tissues. Once LPS binds to TLR4, two signaling pathways are activated: a MyD88-dependent pathway and a MyD88-independent pathway [30, 31]. Based on studies using macrophages, these pathways are responsible for the expression of proinflammatory cytokines [32–34]. Clinical and experimental studies indicate that TLR4 plays a significant role in connecting inflammation and cancer invasion and progression, but the exact mechanism is still not clear. The chemokine CXCL12/SDF-1 and its receptor, CXCR4, have been implicated in invasion, survival, and proliferation of carcinoma cells [35]. Recently, CXCR7 was identified as a second receptor for CXCL12 [36, 37]. Though, some results have indicated that CXCR7 functions as a decoy receptor [38], growing evidence suggested that CXCR7 significantly increases cell proliferation and elevates cellular adhesion property in some conditions [36, 38–41]. We observed that CXCR7 induced by LMW-HA could promote metastasis of PTC cell line W3. However, LMW-HA had no effect on CXCR4 expression. In animal model, differences in CXCR7 expression in tumor masses between the two groups were statistically significant. In addition, the incidence of lymph node metastasis and distant metastasis tended to be higher in patients with PTC with high rather than low expression of TLR4 or CXCR7. Conclusively, the LMW-HA/TLR4/CXCR7 pathway is involved in the development of PTC, suggesting that LMW-HA/TLR4 signaling may be an effective immunomodulatory therapeutic target in PTC.

## 5. Conclusion

In conclusion, we demonstrated that LMW-HA could promote the development of PTC. After binding to TLR4, LMW-HA activated TLR4 signal pathway to promote PTC cell proliferation and migration through upregulation of CXCR7 expression. We also suggested that aberrant expression of TLR4 and CXCR7 in PTC was associated with poor progression of PTC. Therefore, taking the LMW-HA/TLR4/CXCR7 pathway as the potential immunomodulatory therapy target may be a promising approach for PTC treatment.

## Supplementary Material

Supplementary Figure: LMW-HA has no effect on TLR4 negative K1 cell proliferation and migration. To investigate if LMW-HA promotes proliferation and migration of W3 cells via TLR4, TLR4 negative K1 cells was stimulated with LMW-HA, and the data showed that LMW-HA did not promote the proliferation and migration of K1 cells, moreover it also did not upregulate CXCR7 expression on K1 cells.

## Figures and Tables

**Table 1:** Correlation of TLR4 and CXCR7 expression with clinicopathologic features in PTC.

Clinicopathologic parameters	Case no.	TLR4 expression		*P* value	CXCR7 expression		*P* value
		Low	High		Low	High	
Total cases	135	64	71	53%	102	33	24%
Age							
≤60	55	27	28	*P* = 0.745	38	19	*P* = 0.080
* *>60	80	37	43		64	15	
Tissue type							
Normal tissue	56	55	1	*P* = 0.000	55	1	*P* = 0.000
Carcinoma	135	64	71		102	33	
Sex							
Male	65	33	32	*P* = 0.451	48	17	*P* = 0.656
Female	70	31	39		54	16	
Tumor size							
≤5 cm	58	39	19	*P* = 0.000	53	5	*P* = 0.000
>5 cm	77	25	52		49	28	
TNM stage							
I	4	4	0	*P* = 0.000	4	0	*P* = 0.005
II	65	39	26		57	8	
III	38	7	31		24	14	
IV	28	14	14		17	11	
Histologic grade							
I	8	7	1	*P* = 0.000	8	0	*P* = 0.002
II	109	56	53		86	23	
III	18	1	17		8	10	
Lymph nodemetastasis							
Negative	76	50	26	*P* = 0.000	70	6	*P* = 0.000
Positive	59	14	45		32	27	
Distant metastasis							
Negative	108	56	52	*P* = 0.039	89	19	*P* = 0.000
Positive	27	8	19		13	14	

**Table 2:** CXCR7 expression in W3 cell transplanted tumor tissues of nude mice.

Group	*n*	CXCR7 expression		*P* value
		Low	High	
LMW-HA	6	2	4	*P* = 0.002
DMSO	6	6	0	
