# Supplementary material for: Stimulation of TLR4 by LMW-HA Induces Metastasis in Human Papillary Thyroid Carcinoma through CXCR7
Source: Clin Dev Immunol. 2013 Dec 2;2013:712561. doi: 10.1155/2013/712561 (PMC3865734; doi:10.1155/2013/712561)
Supplement: Supplementary file 1 — Supplementary Figure: LMW-HA has no effect on TLR4 negative K1 cell proliferation and migration. To investigate if LMW-HA promotes proliferation and migration of W3 cells via TLR4, TLR4 negative K1 cells was stimulated with LMW-HA, and the data showed that LMW-HA did not promote the proliferation and migration of K1 cells, moreover it also did not upregulate CXCR7 expression on K1 cells. [file 712561.f1.pdf]

Supplemental Figure

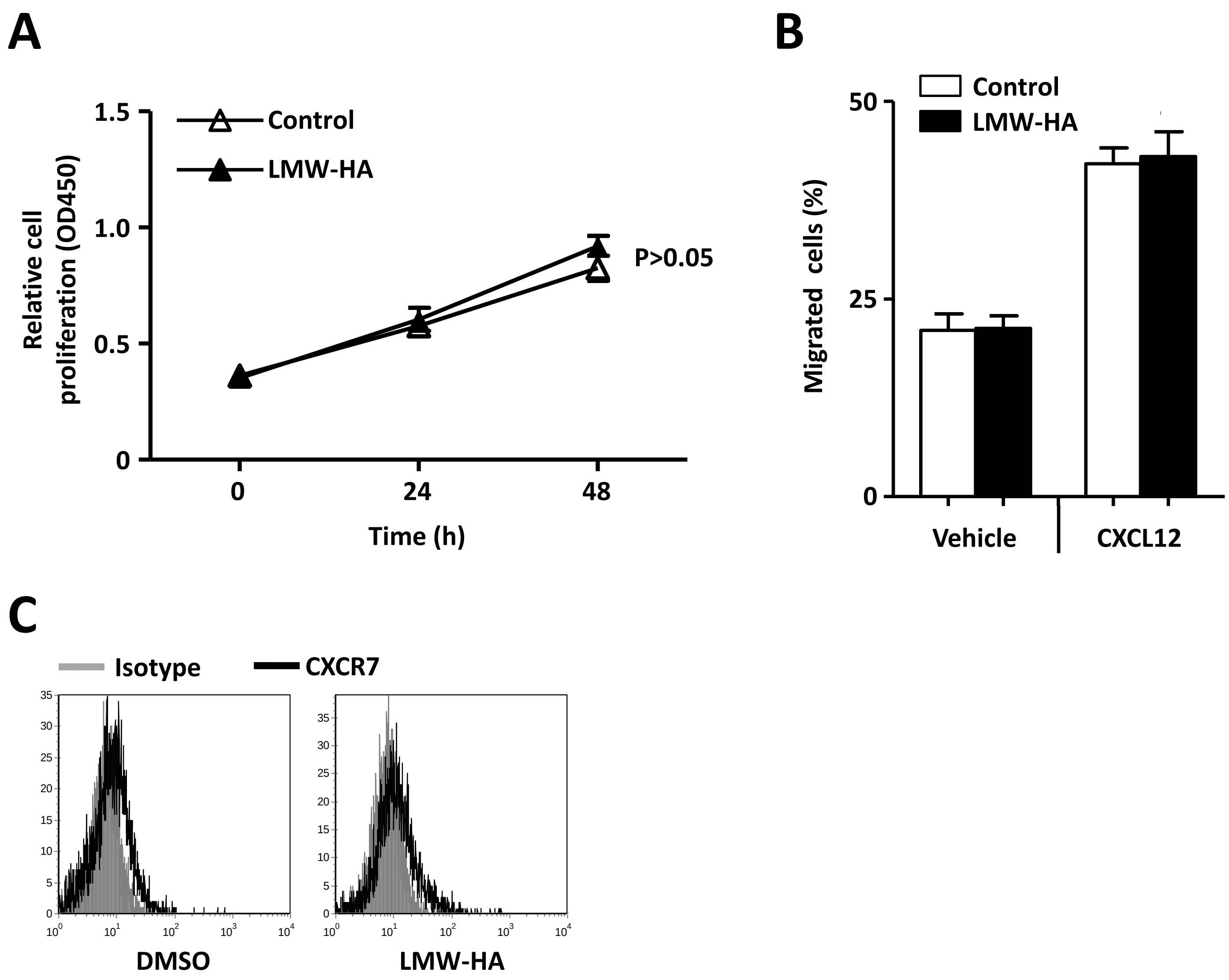

LMW-HA has no effect on K1 cell proliferation and migration. (A) K1 cells were seeded into 96 well plates (2,000 cells/well) and treated with or without LMW-HA. Cell proliferation was analyzed with WST-1 Kit. Data are mean ± SEM for three independent experiments. (B) K1 cells were seeded into the upper chambers of transwell inserts treated with or without LMW-HA, and in the presence or absence of CXCL12 in the lower chambers. Migrated cells were determined. Data are mean ± SEM for three independent experiments. (C) K1 cells were incubated with LMW-HA (100 μg/ml) for 24 h, representative flow cytometric analysis of CXCR7 expression was shown.
